# Shanyou 63: an elite mega rice hybrid in China

**DOI:** 10.1186/s12284-018-0210-9

**Published:** 2018-04-09

**Authors:** Fangming Xie, Jianfu Zhang

**Affiliations:** 1 Yuan Longping High-Tech Agriculture Co. Ltd., Changsha, 410001 Hunan China; 2 Rice Research Institute, Fujian Academy of Agricultural Sciences, Fuzhou, 350018 China

**Keywords:** Hybrid rice, Shanyou 63, Heterosis

## Abstract

Hybrid rice has been successfully used for commercial rice production for 40 years in China. Shanyou 63, a mega rice hybrid, derived from the parents Zhenshan 97A and Minghui 63, was a milestone for China’s hybrid rice development and production because of its high yield and wide adaptability. It was planted in 16 provinces of the country on 17% of the national hybrid rice area annually during the 29 years from 1984 to 2012. The hybrid and its parents have also been widely used for basic and agronomic studies related to rice heterosis, stress tolerance, molecular markers and genomics. We review the development of the hybrid and its parents and their major characteristics for the purpose of learning from the history and guiding future hybrid rice development. The history and development experience show that a successful hybrid rice variety should have multiple traits, including high yield, wide adaptability, resistances to major diseases, and high rice quality that meets the demands of consumers. From the breeding aspect, hybrid rice provides the advantage of combining elite traits or genes from different types of parents, such as those from subspecies of *indica* and *japonica*, into a single variety. Farmers prefer not only a variety with high yield potential, but also stable yields and local adaptability.

## Review

Application of hybrid heterosis in commercial rice production succeeded in 1974 when the essential genetic resources of the 3-line system (cytoplasmic male sterile or CMS, maintainer, and restorer lines) were successfully developed (Yuan and Virmani, 1988). Rice is a self-pollinated crop, which makes commercial hybridization difficult. The development of three lines provides a solution for commercial hybrid seed production through pollinating the CMS line with its corresponding maintainer to produce more CMS seeds, and hybridizing with a restorer line to produce commercial F1 hybrid seeds.

The first generation of 3-line rice hybrids was based on a wild abortive (WA) cytoplasm which was found to be a natural mutation from a wild rice (*O. sativa f. spontanea*) in Hainan, China (Lin and Yuan, 1980). The male parents of the first-generation hybrids were mostly imported from the International Rice Research Institute (IRRI), Philippines and these inbred rice varieties were used directly for producing hybrid combinations. Those inbred lines, including Taiying 1, IR24, IR661, IR26 and IR36, are *indica* germplasm with a good fertility restoration for the hybrids derived from the WA cytoplasmic male sterile (CMS) lines. However, these restorer lines (R-lines) had some obvious weaknesses such as low genetic divergence among themselves, which resulted in stagnant yield increase of the hybrids; lack of adaptation for various rice eco-systems and cropping systems in China, since all were from tropical Asia; and long growth duration because of photoperiod sensitivity. To further improve the R-lines, Chinese scientists started to develop locally adapted male parents using IRRI-developed inbred varieties as donor parents for the characteristics or genes of fertility restoration, grain quality, and disease and insect resistances (Wu et al., 2011). The parents used for the purpose included IR24, IR26, IR30, IR36, IR54, IR64, IR72, IR661, Milyang 46, and IR9761-19. The hybrids generated from those newly-improved male parents, such as Minghui 63 (MH63), Gui 33, and 26 Zhaizao, were significantly improved for hybrid yield and disease resistances, and rapidly increased hybrid rice production in China. MH63 is one of the most successfully developed male parents and played a key role for the promotion of hybrid rice production in China from the late 1980s.

Shanyou 63 (SY63) is the most widely cultivated hybrid rice ever. Both parents and the hybrid were milestones for the renewal of hybrid rice with strong superiority for yield, yield heterosis and wide adaptability. We review the historical development of the parents and the hybrid and describe their major characteristics with the purpose of learning from this history for further hybrid rice development.

## Breeding history

**Fig. 1:**
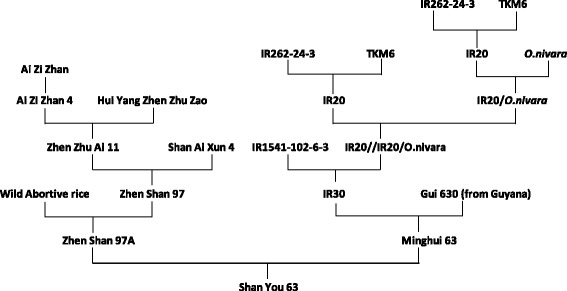
Pedigree of the hybrid rice variety SY63

SY63 is the rice hybrid derived from the female parent Zhenshan 97 A (ZS97A), a WA CMS line, and the male parent MH63 (Fig. 1). ZS97A was developed by continuous backcrosses of the inbred variety ZS97, a semi-dwarf variety developed in Jiangxi province of China for the early-season rice cropping, into a WA plant. The restorer line MH63 was derived from the cross of IR30×Gui 630. IR30 is an IRRI-bred semi-dwarf variety which is a restorer line for WA CMS A-lines with good plant type, high resistance to blast, bacterial blight, and brown planthoppers. The other parent of MH63 is Gui 630, which is an imported rice germplasm from Guyana with the traits of high grain weight, desirable grain quality and high yield potential (Xie et al., 1987).

The cross of IR30×Gui 630 was made in the spring of 1978 at Yacheng of Hainan by the breeder from Sanming Institute of Agricultural Sciences, Fujian (Xie, et al. 1987). The F_1_ plants of the cross were grown in Sha county of Fujian and the progenies were selected using the pedigree method and shuttle breeding between Hainan and Fujian. Hybridization of testcrosses with the female parent of ZS97A started from the F_4_ individual plants in the winter of 1979 and one line (designated E6) among three F_5_ families (E6, E7 and E8) was selected and advanced to F_7_. During the generation advancement, all selected progenies were screened for combining ability of yield and blast resistance, both in the induced nurseries with multiple pathogen races and the field. The final line selected had a high resistance to blast, large panicle and grains, good plant type with erect and thick flag leaf, and maturity 3 days longer than IR24, which is an elite and popular restorer line for hybrid rice based on WA CMS cytoplasm. After different evaluations of combining abilities with different CMS A lines and various yield trials from 1980 to 1981, E6 was finally selected and designated as MH63 and the hybrid combination of ZS97A×MH63 was named as SY63 (Xie, et al. 1987). Since then, it went through various regional, provincial and national yield trials from 1982 to 1985. It performed well with grain yield and blast resistance with an average yield of 7325 kg/ha, 16.2% higher than the yield of hybrid check varieties.

## Adoption and impact

**Fig. 2:**
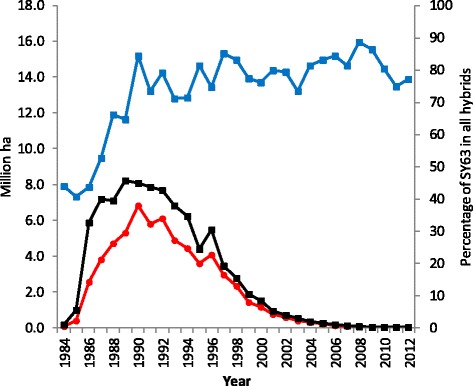
Planting area of SY63 and its percentage as a total of all hybrid rice from 1984 to 2012 in China (Data source: Chinese Ministry of Agriculture and Wu, et al. 2011). Legend

SY63 combines the complementary advantages of its two parents and shows the superior agronomic traits including yield, resistance, wide adaptability and grain quality. It was the most widely cultivated hybrid variety in China over the past four decades. Data collected from the Chinese Ministry of Agriculture showed that SY63 had a large planting area during the years from 1985 to 2001 (Fig. 2) with an average area of 3.6 million hectares and 28.3% of the national hybrid rice growing areas annually. The hybrid was grown on the largest area in 1990, with 6.8 million hectares accounting for 44.8% of the total hybrid rice area in the country. SY63 had been grown in China on a cumulative 62.9 million hectares from 1984 to 2012 with an economic value of 3774 million US dollars corresponding to the increased grain production of 18.8 million tons based on a calculation of average yield increase of 300 kg/ha over check varieties and the rice grain priced as $0.2/kg.

**Fig. 3:**
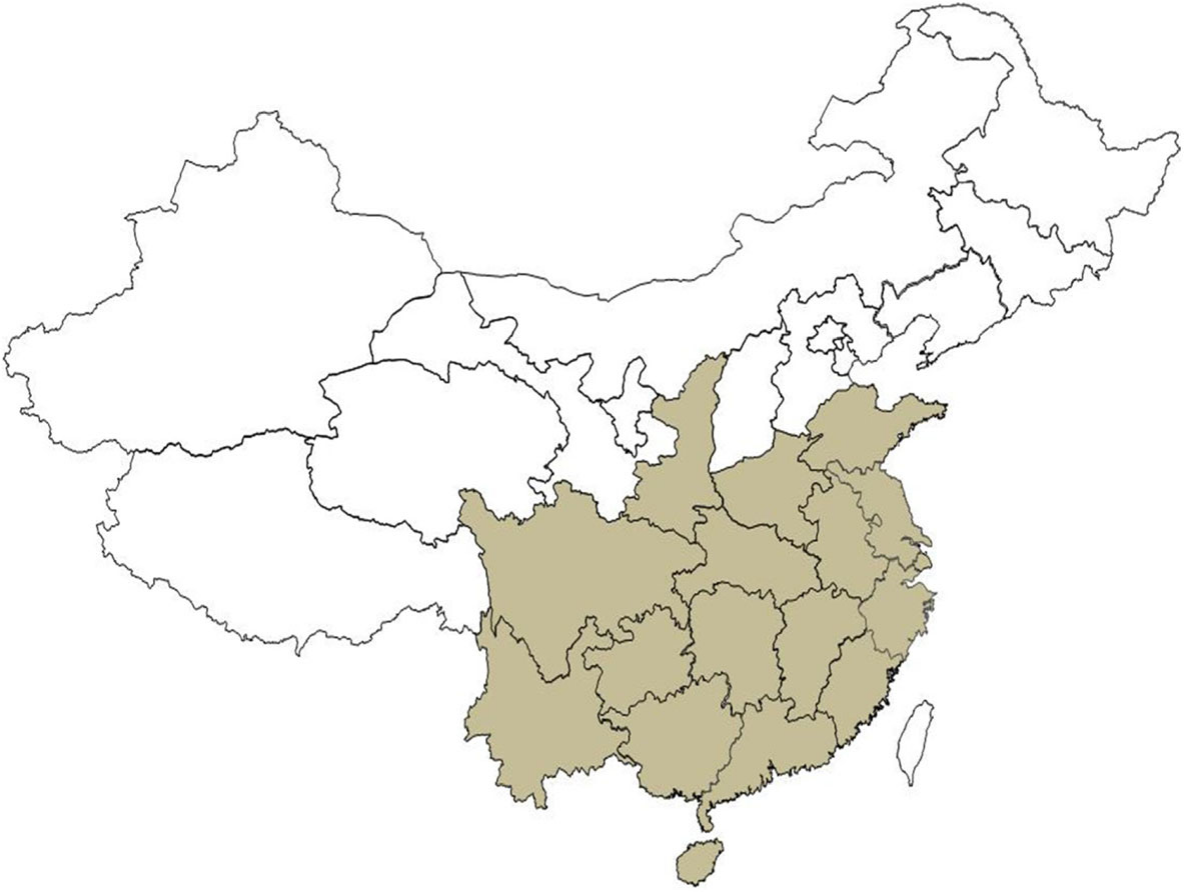
Provinces where SY63 is planted on a significant area in China (colored)

The development of SY63 was regarded as a milestone for the renewal of hybrid rice with strong superiority and wide adaptability. The hybrid was approved for commercial rice production in 16 provinces of central and southern China, ranging from 100^0^ 36′ E (Yunnan) to 121^0^ 56′ E (Shanghai) and from 17^0^ 36’ N (Hainan) to 37^0^ 49′ N (Shandong) geographically, across 21.3 degrees of longitude and 20.2 latitude (Fig. 3).

SY63 has not only the traits of high yield, acceptable grain quality, blast resistance and wide adaptability, but also is suitable for cultivation as a ratoon rice. It accounted for up to 85% of the total ratoon rice in the ratoon rice cropping areas in China from 1998 to 2005 with an accumulated area of 4 million hectares.

## Major genetic and genomic characteristics

SY63 is a semi-dwarf hybrid with an average plant height of 100 to 110 cm at maturity. It has a weak sensitivity to photoperiod and temperature with total growth duration of about 138 days from seed to seed.

The success of SY63 is mostly attributed to its male parent MH63, which is an elite *indica* variety that combines the traits of high yield, good quality, stress resistance and wide adaptability. SY63 yields high because of many favorable QTLs bred into the two parents. MH63 possesses a major gene for yield, *Ghd7*, which controls traits of grains per panicle, plant height and heading date (Xue et al. 2008). Several yield-related QTLs were also mapped on MH63, such as *yd1a*, *yd1b* and *yd2* for grain yield (Yu et al. 1997; Xing et al. 2002), *gp1b* and *gp5* for grains per panicle (Yu et al. 1997), and *gw7*, *gw11*, *TGW3a* and *TGW3b* for 1000-grain weight (Yu et al. 1997; Liu et al. 2010).

During the breeding process, the candidate line of MH63 was screened for resistance to blast disease (*Magnaporthe grisea*). Yang et al. (2008) reported that MH63 has a major QTL, *rbr2*, for blast resistance which is an allele of the *Pib* gene. Wang et al. (2006) cloned a dual functional rice disease resistance-responsive gene from a MH63 cDNA library, and the repressed expression of *OsDR8* showed reduced resistance to bacterial blight (*Xanthomonas oryzae* pv. *Oryzae*) and blast. Another gene, *OsWRKY13* was also cloned from MH63 cDNA library and its overexpression can enhance rice resistance to bacterial blight and blast fungus at both seedling and adult stages (Qiu et al. 2007).

MH63 is moderately resistant to bacterial blight disease. In addition to the two aforementioned dual functional resistance genes *OsWRKY13* and *OsDR8*, *Xa25*(t) for race-specific resistance and *Xa3*/*Xa26* for broad-spectrum resistance were mapped in MH63 (Chen et al. 2002).

MH63 is relatively resistant to sheath blight. Zhang et al. (2008) reported that MH63 had the top resistance level of 2.19, and was the most resistance variety in the selected rice materials among 17 hybrid rice core parents for southern China. Han et al. (2002) identified two QTLs, *qSB-5* and *qSB-9* for sheath blight resistance from MH63.

MH63 is tolerant to potassium deficiency. Peng et al. (2002) reported that MH63 was the most potassium-tolerant variety based on evaluation of plant height, and dry weight of seedling and root.

MH63 is considered insensitive to heat. Fu et al. (2011) found that MH63 was the most tolerant variety to high temperate in a total of frequently-used 39 restorer and maintainer lines at flowering stage.

MH63 is moderately tolerant to salt stress based on evaluation at 5 different NaCl concentrations (Wang et al. 2004).

Hybrid SY63 has intermediate amylose content and a gel consistency of 77.4 mm (Table 1). The cooking and eating qualities are both acceptable to the Chinese consumers.

**Table 1:** Grain quality of SY63 and its parents

Variety	Brown rice	Head rice	Chalky grain	Chalky degree	Ratio of length/width	Gel consistency (mm)	Amylose content
	(%)	(%)	(%)	(%)			(%)
MH63	79.8	49.1	13.2	12.2	3.04	91.0	16.2
ZS97B	76.3	49.3	97.5	31.5	2.3	58.0	23.0
SY63	80.7	28.3	82..5	23.6	2.5	77.4	23.4

Because of its popularity for hybrid rice production, both the parents and the hybrid have been extensively used for genetics and genomics studies during the past four decades. In the first report of resequencing multiple varieties, both parents of SY63 were used among 20 diverse varieties for resequencing of 100 Mb of the unique fraction of the genome (McNally et al. 2009). It was found that large components of the *japonica* genome were introgressed into both of the parents, specifically, on chromosome 1 of ZS97 and chromosome 6 of MH63. Those components contribute greatly to genes controlling heterosis (Yu et al. 1997; Hua et al. 2002; Li et al. 2008; Li et al. 2008; Zhou et al. 2012; Shen et al. 2014; Zhu et al. 2016). Other studies on the association of QTLs and traits included plant height (Xing et al. 2001; Guo et al. 2002; Yu et al. 2002; Guo et al. 2003; Shen et al. 2014), heading date (Xing et al. 2001; Guo et al. 2002; Guo et al. 2003; Yu et al. 2002; Zhu et al. 2016), fertility-restoring genes (He et al. 2002), seedling traits (Cui et al. 2002; Cui et al. 2002; Cui et al. 2010; Zhu et al. 2016), leaf shape (Li et al. 2008), grain quality (Tan et al. 1999; Tan et al. 2001; Xing et al. 2001; Ge et al. 2005; Zheng et al. 2008a; Zheng et al. 2008b), yield-related traits (Yu et al. 1997; Guo et al. 2002; Hua et al. 2002; Xing et al. 2002; Cui et al. 2003; Guo et al. 2003; Xue et al. 2008; Li et al. 2008; Xing et al. 2008; Liu et al. 2010; Liu et al. 2010; Zhou et al. 2012), disease resistance (Han et al. 2002; Chen et al. 2002; Yang et al. 2003; Li et al. 2010; Kou et al. 2010), tolerance of nitrogen deficiency (Lian et al. 2005; Wei et al. 2012), and drought tolerance (Lian et al. 2005; Wei et al. 2012).

In a new study for rice heterosis, Zhang et al. (2016) performed map-based sequencing of both parents of SY63 with coverages of 90.6% (ZS97B) and 93.2% (MH63) of estimated genome sizes. Comparative analyses of these two *indica* genomes uncovered surprising structural differences, specifically with respects to inversions, translocations, presence/absence variations, and segmental duplications. The number of genes expressed in the hybrid was higher by 1059–2217 than those of parents in three tissues of seedling shoot, panicle and flag leaf, but also very different from the parents, which shows a complementary gene action between the parental genomes for superior field performance.

Both the parents and the hybrid are the most widely used materials for genetic and genomic research. Genomes of both parents have been resequenced for further studies of heterosis and other agronomic traits and associations of genes and traits (McNally et al. 2009; Xu et al. 2014; Zhang et al. 2016; Zhang et al. 2016).

## Important progeny

Because of its excellent traits of strong restoring ability and capacity of restoring fertility to a wide range of CMS female parents, good combining ability and disease resistance, MH63 is not only an excellent combiner with ZS97A, but it has also been used to develop hybrids using CMS A-lines of different sterile cytoplasms, such as Dian, Gam, Indonesia Paddy, Honglien, and Dwarf Abortion. A total of 34 hybrids at provincial level and 4 hybrids at national level were approved for commercial production up to 2014 with different cytoplasmic A-lines other than WA cytoplasm. Recently, MH63 has also even been used as male parents for two-line hybrids based on themo- or photo-sensitive genic male sterile lines. Statistics showed that the planting area of hybrids using MH63 as male parent has cumulatively reached 84.5 million hectares and accounted for 20.6% of the total hybrid rice in China from 1984 to 2014.

The hybrids derived from MH63 were also being planted in other countries such as Myanmar, Vietnam, India, Laos and Cambodia with good performance in demonstration trials (Wu et al. 2011).

At the same time, MH63 has been used greatly in hybrid rice breeding programs for improving R-lines. It is the most widely used germplasm contributing to the improvement of hybrid rice male parents in China. Data showed that a minimum of 594 new restorer lines were developed from MH63 with 978 hybrids approved for commercial rice production at the provincial level, and 170 at the national level up to 2010. From 1990 to 2015, the hybrids with male parents that were progenies of MH63 were planted on 105 million hectares, which was 28.7% of the total national hybrid rice area.

## Conclusions

The success of development of the hybrid rice cultivar SY63 led to increased adoption of hybrid rice by the Chinese farmers. MH63, the male parent of SY63, played a key role for the hybrid rice because of its well-combined traits of high yield, resistance to diseases and insects, and adaptability to diverse growing environments. The planting area of SY63 started to gradually decline from the 1990’s because better and high-yielding hybrids based on the two-line hybrid rice system are developed and high quality of rice is more required by consumers. Also, as more and better rice varieties are being developed, farmers have more opportunities of choices for a hybrid variety based on yield, disease resistance, location adaption, as well as grain quality. However, MH63 has still been used widely for development of male parents for two-line hybrids because of its excellent agronomic, yield and disease resistance traits. Since SY63 was developed, no other hybrid rice variety has been planted at such a large scale. SY63 has contributed tremendously to the security of the food supply in China, and has greatly promoted hybrid rice development.
